# Epidemiological indices with multiple circulating pathogen strains

**DOI:** 10.1016/j.idm.2025.03.006

**Published:** 2025-03-17

**Authors:** Cristiano Trevisin, Lorenzo Mari, Marino Gatto, Vittoria Colizza, Andrea Rinaldo

**Affiliations:** a Laboratory of Ecohydrology, École Polytechnique Fédérale de Lausanne, Station 2, Lausanne, 1015, Switzerland; b Sorbonne Université, INSERM, Pierre Louis Institute of Epidemiology and Public Health, 27 rue Chaligny, Paris, 75012, France; c Dipartimento di Elettronica, Informazione e Bioingegneria, Politecnico di Milano, Via Ponzio 34/5, Milano, 20133, Italy; d Dipartimento di Ingegneria Civile, Edile e Ambientale (ICEA), Università di Padova, Via Marzolo 9, Padova, 35131, Italy

**Keywords:** COVID-19, Epidemic control, Variants, Reproduction numbers, Epidemicity indices, Infectious diseases

## Abstract

Epidemiological indicators (e.g. reproduction numbers and epidemicity indices) describe long- and short-term behaviour of ongoing epidemics. Their evolving values provide context for designing control measures because maintaining both indices below suitable thresholds warrants waning infection numbers. However, current models for the computation of epidemiological metrics do not consider the stratification of the pathogen into variants endowed with different infectivity and epidemiological severity. This is the case, in particular, with SARS-CoV-2 infections. Failing to account for the variety of epidemiological features of emerging variants prevents epidemiological indices from spotting the possible onset of uncontrolled growth of specific variants, thus significantly limiting the prognostic value of the indicators. Here, we expand an existing framework for the computation of spatially explicit reproduction numbers and epidemicity indices to account for arising variants. By analysing the data of the COVID-19 pandemic in Italy, we show that embedding additional layers of complexity in the mathematical descriptions of unfolding epidemics reveals new angles. In particular, we find epidemiological metrics significantly exceeding their thresholds at the emergence of new variants. Such values foresee a recrudescence in new infections that only becomes evident after emerging new variants have effectively replaced the previous active strains. The demography of the variant composition flags the presence of specific strains growing more rapidly than the total number of infections generated by all variants combined. Variant-aware epidemiological indicators thus allow to engineer better control measures tailored to the shifting patterns of severity and evolving features of infectious disease epidemics.

## 1 Introduction

After its emergence in late 2019, the virus responsible for the COVID-19 pandemic, SARS-CoV-2, has undergone many mutations. A major effort has been devoted to stemming the spread of the virus after the pandemic outbreak—via both vaccines and non-pharmaceutical interventions (Bertuzzo et al., 2020; Colizza et al., 2021; Lemaitre et al., 2022; Leung et al., 2023)—generated strong evolutionary pressure on the virus to evade immunity conferred by prior vaccination and infection (Carabelli et al., 2023; Grabowski et al., 2022; Koelle et al., 2022). In particular, newly emerging strains achieved higher reproduction numbers, shorter doubling times (Goldberg et al., 2023; Koelle et al., 2022) and reduced their mean generation time (Kim et al., 2022; Zhang et al., 2021). This pattern has not only affected significantly the course of SARS-CoV-2, but also that of other infectious diseases such as dengue fever and influenza (Guzman & Harris, 2015; Lin et al., 1999). Several studies have included variant stratification in their compartmental models to understand the effect of control measures (Saviano et al., 2023; Cuevas-Maraver et al., 2024) or to predict the course of an epidemic (Baccega et al., 2024) in the presence of co-circulating strains. Another study (Sabbatini et al., 2024) accounted for the effect of the variant prevalence to estimate the exposure and the hospitalization rates.

The fate of an epidemic is recapitulated by the computation of effective prognostic indicators. Foremost among them is the reproduction number, R, which measures the number of secondary infections produced by each primary infector within a given community. Values of R above the unity flag an accelerating spread of infections, whereas subunit values characterize an asymptotic waning epidemic because no endemic equilibrium may be established. In practice, reproduction numbers command stricter or milder imposed restrictions via the enforcement of control measures aimed at achieving subunit values of R. Depending on the chosen epidemiological modelling framework, R may be computed via next-generation matrices, whenever a SIR-like compartmental model is adopted (Diekmann et al., 2010; Van Den Driessche, 2017), or via renewal equations (Cori et al., 2013) that allow one to compute reproduction numbers directly from data.

Another key indicator is the epidemicity index (Mari et al., 2018; Neubert & Caswell, 1997; Trevisin et al., 2022, 2024) pinpointing the largest amplification attained in the short-term by any impulsive perturbation to a disease-free equilibrium (Mari et al., 2017, 2018, 2021, 2024). Notably, the asymptotic stability condition R<1 does not prevent local transient outbursts of infections (Mari et al., 2024). It has been formally demonstrated that excluding the possibility of short-term flare-ups requires R substantially lower than its endemicity threshold (R≥1) (Mari et al., 2024; Trevisin et al., 2022, 2024). This questions the prognostic power of reproduction numbers alone to sort out the most effective control measures during in times of spreading infections.

Within a metapopulation, infections may be transmitted among spatially connected communities via e.g. contacts driven by human mobility. The COVID-19 pandemic highlighted the relevance of spatial connectivity in both our understanding of the spread of SARS-CoV-2 towards proper identification of control measures. Spatial formulations of the reproduction number (Birello et al., 2024; Trevisin et al., 2023) and the epidemicity index (Trevisin et al., 2024) were derived. Results highlighted how incorporating a spatial stratification improves the estimates of R, in particular because they account for export/import of infections within connected communities, allowing a more precise identification of appropriate control measures.

Another stratification, pertaining to the emergence of different variants of the virus of interest, is still missing to date. We posit that embedding strain diversification in a framework for the computation of epidemiological indices and looking at anomalies in their temporal patterns sheds light on the behaviour of new variants. To test this hypothesis, we propose to expand the existing spatially explicit model for the computation of epidemiological metrics (Mari et al., 2024; Trevisin et al., 2024) to provide a comprehensive framework that seamlessly derives, in real-time, space- and variant-specific values of both the reproduction number and the epidemicity index. This is claimed to allow us to single out the fastest-growing strain within the total number of new infections, thus anticipating possible recrudescence in new infections. Our approach differs from previous attempts at incorporating variant stratification into compartmental models as we do not calibrate a compartmental model but rather infer time-series of epidemiological indices that may serve as a basis for control measures. While our framework requires one to know the variants’ generation times in advance, it does not require specific assumptions for direct transmission, and may be computationally lighter than compartmental approaches.

## 2 Methods

We propose a framework based on a Leslie projection matrix (Mari et al., 2024; Trevisin et al., 2024), here expanded to account for variant emergence. A key assumption is that once a variant has emerged, it is assumed to act independently of the other circulating strains. Moreover, any interaction among variants (notably competition) is subsumed by their respective R and generation time distributions (*β*(*τ*) i.e., the time elapsed between the infector's exposure and that of their infectee(s)).

Let us consider a metapopulation composed of *N* interconnected human communities (or nodes), where several variant genotypes (*v* = 1, …, *V*) of a generic infectious disease are circulating. The compartment infected by strain *v* in an arbitrary community *j*(*j* = 1, …, *N*) is indicated as $I_{j}^{v}\left(t,\tau \right)$, a variable of both time *t* and age of infection *τ*. Also, let *C*_*lj*_(*t*) be the proportion of residents of the community *j* who commute daily to *l* for their daily activities (Trevisin et al., 2023). The following differential problem holds (Mari et al., 2024; Trevisin et al., 2023):

(1a) $$\frac{\partial I_{j}^{v}\left(t,\tau \right)}{\partial t}+\frac{\partial I_{j}^{v}\left(t,\tau \right)}{\partial \tau }=-\gamma^{v}\left(\tau \right)I_{j}^{v}\left(t,\tau \right)$$

(1b) $$I_{j}^{v}\left(t,0\right)=\overset{N}{\underset{k=1}{\sum }}z_{jk}\left(t\right)R_{k}^{v}\left(t\right)\int_{0}^{\infty }\phi^{v}\left(\tau \right)I_{k}^{v}\left(t,\tau \right)d\tau \;\forall t\;\left(B.C.\right),$$

where *γ*^*v*^(*τ*) represents the instantaneous rate of exit from the infected compartment (because of recovery, death, quarantine, or isolation), and *ϕ*^*v*^(*τ*) is the rate of secondary transmission per single infectious case. Regardless of the variant being considered, the elements *z*_*jk*_ are defined as

(2) $$z_{jk}\left(t\right)=\overset{N}{\underset{l=1}{\sum }}\frac{C_{lj}\left(t\right)n_{j}}{\sum_{m=1}^{N}C_{lm}\left(t\right)n_{m}}C_{lk}\left(t\right),$$

denoting each node's exposure to infectious individuals residing in a different one via several spatial routes of infection. Both matrices **C** = [*C*_*lj*_] and **Z** = [*z*_*lj*_] are column-stochastic. The rate of secondary transmission *ϕ*^*v*^(*τ*) relates to the distribution of generation times, *β*^*v*^(*τ*), via the following relation:

(3) $$\beta^{v}\left(\tau \right)=p^{v}\left(\tau \right)\phi^{v}\left(\tau \right),$$

where $p^{v}\left(\tau \right)=\exp\left(-\int_{0}^{\tau }\gamma^{v}\left(\xi \right)d\xi \right)$ represents the fraction of infected individuals that are still infectious *τ* days after infection.

To comply with a daily or weekly collection of epidemiological data, one may discretize the above process with respect to both the time and the age of infection, which leads to the following system of finite-difference equations:

(4a) $$I_{j,1}^{v}\left(t\right)=\sigma_{0}^{v}\sum_{k=1}^{N}z_{jk}\left(t\right)R_{k}^{v}\left(t\right)\sum_{i=1}^{q}f_{i}^{v}I_{k,i}^{v}\left(t-1\right)$$

(4b) $$I_{j,i}^{v}\left(t\right)=\sigma_{i-1}^{v}I_{j,i-1}^{v}\left(t-1\right)\;\;\;for\;i\geq 2,$$

where $f_{i}^{v}=\int_{i-1}^{i}\phi^{v}\left(\tau \right)d\tau$ is a discretization in time of function *ϕ*^*v*^(*τ*), and $\sigma_{i-1}^{v}=\frac{p^{v}\left(i\right)}{p^{v}\left(i-1\right)}$ represents the proportion of infectious individuals with age of infection *i* − 1 still infectious on the next day. Inference of terms $R_{k}^{v}\left(t\right)$ is possible via sequential Monte Carlo algorithms (Trevisin et al., 2023).

### 2.1 The multi-strain leslie projection matrix

Let us rewrite process (4) in matrix form and define a column vector **I**(*t*) collecting all the infectious compartments at time *t*, that is:

(5) $$I\left(t\right)=\overset{V}{\underset{v=1}{⋃}}I^{v}\left(t\right),$$

with:

(6) $$I^{v}\left(t\right)={\left\{I_{1,1}^{v},\ldots ,I_{N,1}^{v},\ldots ,I_{1,q}^{v},\ldots ,I_{N,q}^{v}\right\}}^{T},$$

where *T* indicates matrix transposition.

The projection matrix associated with model (4) is defined such that **I**^*v*^(*t* + 1) = **L**^*v*^(*t*)**I**^*v*^(*t*). The non-zero block **L**^*v*^(*t*) for a given variant strain *v* has the structure of a so-called Leslie projection matrix and reads

(7) $$L^{v}\left(t\right)=\left(\begin{array}{lllll} T_{1}^{v}\left(t\right) & T_{2}^{v}\left(t\right) & \cdots & T_{q-1}^{v}\left(t\right) & T_{q}^{v}\left(t\right)\\ \sigma_{1}^{v}1_{N} & 0_{N} & \cdots & 0_{N} & 0_{N}\\ 0_{N} & \sigma_{2}^{v}1_{N} & \cdots & 0_{N} & 0_{N}\\ \vdots & \vdots & \ddots & \vdots & \vdots \\ 0_{N} & 0_{N} & \cdots & \sigma_{q-1}^{v}1_{N} & 0_{N} \end{array}\right),$$

where **1**_*N*_ and **0**_*N*_ are the identity and null matrix of order *N*, respectively, and the sub-matrices $T_{i}^{v}\left(t\right)$ are defined as:

(8) $$T_{i}^{v}\left(t\right)=\sigma_{0}^{v}f_{i}^{v}\left(\begin{array}{llll} R_{1}^{v}\left(t\right)z_{11}\left(t\right) & R_{2}^{v}\left(t\right)z_{12}\left(t\right) & \ldots & R_{N}^{v}\left(t\right)z_{1N}\left(t\right)\\ R_{1}^{v}\left(t\right)z_{21}\left(t\right) & R_{2}^{v}\left(t\right)z_{22}\left(t\right) & \ldots & R_{N}^{v}\left(t\right)z_{2N}\left(t\right)\\ \vdots & \vdots & \ddots & \vdots \\ R_{1}^{v}\left(t\right)z_{N1}\left(t\right) & R_{2}^{v}\left(t\right)z_{N2}\left(t\right) & \ldots & R_{N}^{v}\left(t\right)z_{NN}\left(t\right) \end{array}\right).$$

Therefore, the first *N* rows of **L**^*v*^(*t*) represent the appearance of new infections. In contrast, each of the $\sigma_{i}^{v}I_{N}$ blocks corresponds to the transition of infectious individuals with age of infection *i* to their *i* + 1-th day of infection.

For multiple circulating variants, the global projection matrix **M**(*t*) is block-diagonal and reads:

(9) $$M\left(t\right)=\left(\begin{array}{llll} L^{1}\left(t\right) & 0_{Nq} & \cdots & 0_{Nq}\\ 0_{Nq} & L^{2}\left(t\right) & \cdots & 0_{Nq}\\ \vdots & \vdots & \ddots & \vdots \\ 0_{Nq} & 0_{Nq} & \cdots & L^{V}\left(t\right) \end{array}\right),$$

with **0**_*Nq*_ being a null matrix of order *Nq*.

### 2.2 Long-term analysis

To assess the long-term behaviour of the system and the feasibility of a disease-free equilibrium, one first has to extract the next-generation matrix (van den Driessche & Watmough, 2002) and its spectral radius from the relevant Leslie projection matrix. The latter is defined as global effective reproduction number (Mari et al., 2021; Trevisin et al., 2024). To yield the next-generation matrix, each diagonal block **L**^*v*^(*t*) of the general projection matrix **M**(*t*) must be split into two matrices, a transmission matrix **T**^*v*^(*t*) and a transition matrix **Σ**^*v*^(*t*), such that **T**^*v*^(*t*) + **Σ**^*v*^(*t*) = **L**^*v*^(*t*). The variant-specific next-generation matrix (Allen & van den Driessche, 2008) then reads

(10) $$K^{v}\left(t\right)=T^{v}\left(t\right){\left(1_{N\times q}-\Sigma^{v}\left(t\right)\right)}^{-1},$$

where **1**_*Nq*_ is the identity matrix of order *Nq*. It is quite straightforward to prove that the following relation holds:

(11) $$K\left(t\right)=\left(\begin{array}{llll} K^{1}\left(t\right) & 0_{Nq} & \cdots & 0_{Nq}\\ 0_{Nq} & K^{2}\left(t\right) & \cdots & 0_{Nq}\\ \vdots & \vdots & \ddots & \vdots \\ 0_{Nq} & 0_{Nq} & \cdots & K^{V}\left(t\right) \end{array}\right).$$

For each variant, its national $R_{v}^{G}$ (i.e., the indicator that predicts the long-term fate of that specific variant in the considered metapopulation) is defined as the spectral radius of the variant-specific next-generation matrix, **K**^*v*^(*t*):

(12) $$R_{v}^{G}\left(t\right)=\rho \left(K^{v}\left(t\right)\right),$$

with *ρ*(⋅) indicating the spectral radius operator. In particular, the condition $R_{v}^{G}\left(t\right)<1$, if maintained for all *t*'s, guarantees that strain *v* cannot establish in the community, thus preventing endemic transmission. Because of the property (11), one can easily conclude that the global $R^{G}$, which encompasses all variants and predicts the fate of the disease as a whole, is the largest of the variant-specific global R:

(13) $$R^{G}\left(t\right)=\underset{v}{\max}R_{v}^{G}\left(t\right).$$

If $R^{G}\left(t\right)<1$ for all *t*'s, no variant can ever establish in the community.

### 2.3 Short-term reactivity analysis

One may also analytically assess whether a short-term flare-up is possible under given epidemiological conditions. Similarly to the long-term analysis, the block-diagonal nature of the Leslie projection matrix brings a substantial simplification of the relevant algebra. As such, one may define the epidemicity index for each variant subset *v* as the maximum one-step growth of the compartments infected with variants *v*, that is:

(14) $$E_{v}\left(t\right)=\underset{\|Y^{v}\left(t\right)\|\neq 0}{\max}\frac{\|Y^{v}\left(t+1\right){\|}_{p}}{\left\|Y^{v}\left(t\right)\right\|}=\underset{\|I^{v}\left(t\right)\|\neq 0}{\max}\frac{\|WL^{v}\left(t\right)I^{v}\left(t\right){\|}_{p}}{\|WI^{v}\left(t\right){\|}_{p}},$$

where ‖*x*‖ = *∑*_*i*_|*x*_*i*_| is the *ℓ*^1^-norm of vector *x*, **I**^*v*^(*t*) is the set of infectious classes (in space and age of infection) for strain *v* at time *t*, **Y**^*v*^ is a customary observed variable based on **I**^*v*^(*t*), and **W** is a suitable transformation matrix. The condition $E_{v}\left(t\right)<1$, if maintained for all *t*'s, prevents the abundance of individuals infected with strain *v* from ever increasing in the short term, even if infected people enter the community. Other algebraic norms (see Trevisin et al. (2024)) are possible, but here we only focus on the *ℓ*^1^-norm because of its straightforward interpretation. The block-diagonal property of the general projection matrix **M**(*t*), allows one to define the global epidemicity index as:

(15) $$E^{G}\left(t\right)=\underset{v}{\max}E_{v}\left(t\right).$$

If $E^{G}\left(t\right)<1$ for all *t*'s, no variant can ever undergo a temporary prevalence increase in the population.

In the following, we discuss the short-term epidemicity of the system through the observed variable **Y**, defined as the vector of length *NV* that contains as elements the absolute abundances of infectious individuals per each variant strain and spatial node. This vector reads

(16) $$Y=\overset{N}{\underset{v=1}{⋃}}Y^{v},\;with\;Y^{v}=\left\{Y_{j}^{v},\;for\;j=1,\ldots ,N\right\},$$

where

(17) $$Y_{j}^{v}=\overset{q}{\underset{i=1}{\sum }}I_{j,i}^{v}.$$

The corresponding transformation matrix **W** such that **Y**(*t*) = **WI**(*t*) takes the form

(18) $$W=\underset{\;\;N\times V\;\text{times}\;\;}{\underbrace{\left(\begin{array}{llll} 1_{q} & 1_{q} & \cdots & 1_{q} \end{array}\right)}},$$

with **1**_*q*_ being a diagonal matrix of order *q*. Computing the epidemicity index applying the *ℓ*^1^-norm to this observed variable highlights the variant-node pairs that could achieve the largest short-term growth in the total number of infected individuals. Other state transformations could be applied as well (see Trevisin et al., 2024) but here we limit our attention to this one out of simplicity.

Given the column-stochasticity of matrix **Z**(*t*) *∀t*, it is possible to derive an explicit formula for the computation of the epidemicity index for variant *v*, namely

(19) $$E^{v}\left(t\right)=\sigma_{0}^{v}\underset{j}{\max}R_{j}^{v}\left(t\right)\underset{i}{\max}f_{i}^{v}+\sigma^{v},$$

with the additional assumptions that $\sigma_{i}^{v}=\sigma^{v}\;\forall i\in \left\{1,\ldots ,q-1\right\}$, indicating a constant rate of exit from the infectious compartments, and $f_{q}^{v}=0$, which underlines negligible infectiousness at the age of infection *τ* ≥ *q*.

Inverting this formula allows one to compute the threshold reproduction number $R_{⋆}^{v}<1$ that should be achieved to guarantee that a specific variant strain is non-reactive (Mari et al., 2024):

(20) $$R_{⋆}^{v}=\frac{1-\sigma^{v}}{\sigma_{0}^{v}\underset{i}{\max}f_{i}^{v}}.$$

Thanks to the block-diagonal property of the Leslie projection matrix, one may easily compute the global epidemicity index as the largest variant-specific one:

(21) $$E^{G}\left(t\right)=\underset{v}{\max}E^{v}\left(t\right)=\underset{v,k}{\max}\left[\sigma_{0}^{v}R_{k}^{v}\left(t\right)\underset{i}{\max}f_{i}^{v}+\sigma^{v}\right].$$

### 2.4 Application to COVID-19

**Fig. 1:**
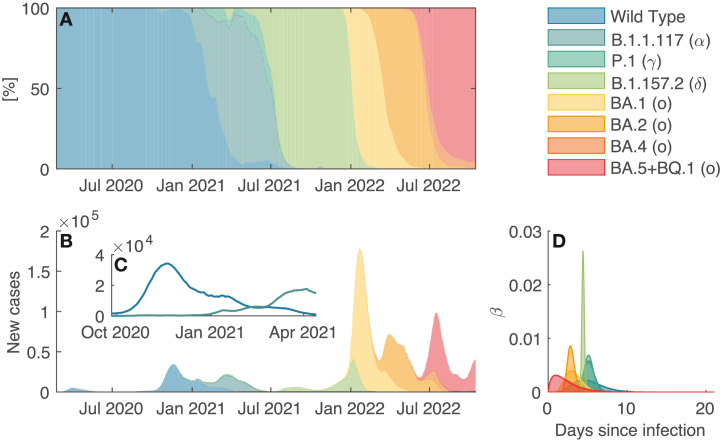
**A**:Variant prevalence for the COVID-19 pandemic in Italy; **B**: estimated number of infections attributable to each variant; **C**: same as in **B**, zoom on wild type and B.1.1.117 strains between end of September 2020 and mid-April 2021; **D**: visual representation of each variant's generation interval distribution.

While this study can be implemented for any directly transmitted infectious disease, we tailor it to the covid-19 pandemic in Italy. The distributions of generation time for the considered SARS-CoV-2 variants are assumed to be gamma-shaped, with details shown in Table 1 and shown in Fig. 1. The depletion of the infectious compartment, regardless of the considered variant, follows a negative exponential distribution, namely *p*(*τ*) = exp(−0.068*τ*), which yields *σ*_*j*_ = exp(−0.068) *∀j* ≥ 1, corresponding to a mean residence time within the infectious compartment of approximately 11.7 days (Gatto et al., 2020). Whenever estimates of the generation times’ distribution are not available, the serial interval is used instead (Chen et al., 2022; Svensson, 2007; Torneri et al., 2021).

**Table 1:** Metrics of the generation time distribution of the considered variants in the multi-variant setup. The mean of the generation time *μ*_*β*_ is given. The standard deviation *σ*_*β*_ is obtained from the confidence intervals. $R_{⋆}^{v}$ computed using Equation (20) and assuming a daily discretization of the generation time distribution are also shown.

Common name	Lineage	*μ*_*β*_ (95 % CI) [days]	*σ*_*β*_ [days]	$R_{⋆}^{v}$	Ref.
Wild type	–	5.20 (4.40–6.00)	1.87	0.23	†
Alpha	B.1.1.117	5.20 (4.87–5.47)	0.70	0.09	‡
Gamma	P.1	5.18 (4.93–5.43)	0.58	0.07	§
Delta	B.1.157.2	4.43 (4.36–4.49)	0.15	0.06	§
Omicron	BA.1	3.30 (2.80–3.70)	1.05	0.14	¶
	BA.2	2.90 (2.70–3.10)	0.47	0.07	¶
	BA.4	2.80 (1.50–6.70)	6.08	0.22	¶
	BA.5+BQ.1	2.30 (1.60–3.10)	1.75	0.19	¶

**References:**^†^Chen et al. (2022), ^‡^Jusot (2022), ^§^Galmiche et al. (2023), ^¶^Madewell et al. (2023).

Daily reported infections in the 20 Italian regions are gathered from the Italian Department of Public Protection (github.com/pcm-dpc/COVID-19). As for human mobility is considered, the connectivity matrix **C** as in (Trevisin et al., 2024) through mobility data collected from the Italian Institute of Statistics (istat.it/it/archivio/139381) and updated via the “Workplace mobility” time series of the Google Community Mobility Reports (google.com/covid19/mobility/). The simulations shown here extend from February 23, 2020, through October 15, 2022. The data on the prevalence of variants in Italy are obtained from the data portal of the European Centre for Disease Prevention and Control (GISAID, see Khare et al. (2021); Elbe and Buckland-Merrett (2017); Shu and McCauley (2017), and TESSy frameworks) and is shown in Fig. 1. The following variants are considered for this study: the wild type, Alpha (B.1.1.117), Gamma (P.1), Delta (B.1.157.2), and Omicron subtypes (BA.1, BA.2, BA.4, BA.5+BQ.1). To contain the computational burden, all other variants subtypes are not considered, as their prevalence among sequenced samples was low at all times. In addition, isolated spurs in the reporting of cases associated with a given variant occurring more than a month apart from that variant's main transmission period are removed to reduce the computational burden.

### 2.5 Numerical experiments

In the following, we compare a variant-specific scenario and a non-stratified one. Concerning the non-stratified scenario, the vector **Y**_1_ has length *N*, and its transformation matrix has size (*q*, *N*). Model (4) loses its dependency on the variant and takes the form (Trevisin et al., 2024)

(22a) $$I_{j,1}\left(t\right)=\sigma_{0}\sum_{k=1}^{N}z_{jk}\left(t\right)R_{k}\left(t\right)\sum_{i=1}^{q}{\hat{f}}_{i}I_{k,i}\left(t-1\right)$$

(22b) $$I_{j,i}\left(t\right)=\sigma_{i-1}I_{j,i-1}\left(t-1\right)\;\;\;\;for\;i\geq 2,$$

where, to allow inter-scenario comparisons, we compute each ${\hat{f}}_{k,i}$ term as the weighted average of the variant-specific $f_{i}^{v}$, with weights equal to the active infections (per node), that is

(23) $${\hat{f}}_{k,i}=\frac{\overset{V}{\underset{v=1}{\sum }}f_{i}^{v}I_{k,i}^{v}\left(t-1\right)}{\overset{V}{\underset{v=1}{\sum }}I_{k,i}^{v}\left(t-1\right)}.$$

## 3 Results

### 3.1 Computation of the variant-specific effective reproduction numbers

**Fig. 2:**
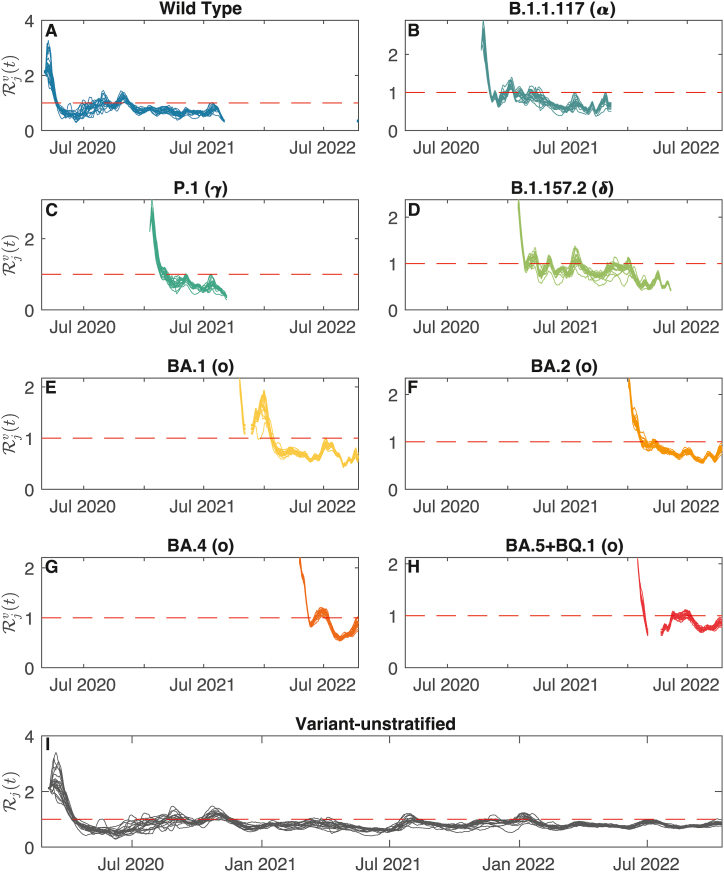
Local effective reproduction numbers for different SARS-CoV-2 strains and different model set-ups during the COVID-19 pandemic in Italy. **A**–**H**: Variant-specific R; **I**: Variant-unstratified R.

The spatially connected effective reproduction numbers (R) of the considered variant strains are computed by running a sequential Monte Carlo algorithm independently for each circulating variant (Trevisin et al., 2023). We show in Fig. 2 both the local variant-specific R for each considered variant and, for the sake of completeness, the local variant-unstratified R (Trevisin et al., 2024). At the beginning of the spread of each strain, the variant-specific R take a value that is substantially larger than the unit threshold—the divide between endemic spread and disease-free conditions—thus indicating a rapid initial spread, where the number of new infections caused by a given variant undergoes exponential growth. After the variant has consolidated, its R decreases and starts oscillating around the unit threshold, a consequence of immunity build-up in the population and control measures. Interestingly, yet unsurprisingly, one may observe that, as soon as a new, fitter variant emerges, the R of the previous ones tend to steadily remain below the unit threshold until the old variants disappear or become undetectable, a purely mathematical consequence of the strain waning. In addition, one may note a larger variability among the local variant-specific values of R for the first strains (wild type, Alpha and Delta) than for later strains. These differences, however, remain small, and all nodes seem to react quite similarly to the circulation of SARS-CoV-2. The variant-unstratified local R, on the other hand, appears much less prone to temporal fluctuations. Specifically, the initial jump observed in the variant-specific reproduction number is not matched by a substantial increase of the variant-unstratified index above the endemicity threshold R=1. Still, the rise in new recorded infections leads to a temporary growth of R above the endemicity threshold (e.g., before January 2021, or after July 2021).

### 3.2 Computation of the national epidemiological indices

**Fig. 3:**
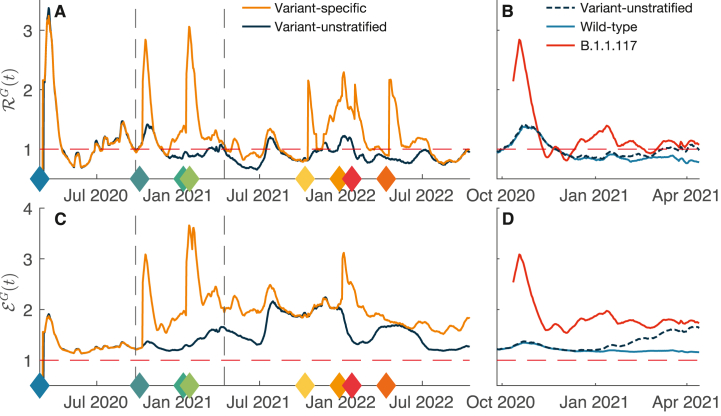
General epidemiological indices evaluated for different model set-ups. **A**: national effective reproduction number; dashed vertical lines highlight the temporal span shown in panel **B**, while filled diamonds mark the emergence (first recorded case) of a new variant and are colour-coded according to Fig. 1. **B**: as in **A**, showing national R for SARS-CoV-2 wild type and B.1.1.117 (here highlighted in red) between the end of September 2020 and mid-April 2021 against the variant-unstratified national R. **C**: as in **A**, but showing the epidemicity index; **D**: as in **C**, but between the end of September 2020 and mid-April 2021.

The national $R^{G}$ and epidemicity index $E^{G}$ are shown in Fig. 3 for both the variant-specific and variant-unstratified scenarios. As previously observed (Fig. 2), whenever a new variant emerges, its local variant-specific R values are characterized by an initial spike. This sudden jump is also seen if the global variant-specific $R^{G}$ is computed, essentially the largest among the national variant-specific values of R (Fig. 3A).

The emergence of a new variant often brings the two time series (variant-specific and -unstratified global reproduction numbers) to the two different sides of the unit threshold, the variant-unstratified denoting disease-free equilibrium conditions. Out of the considered simulation span of 965 days, this occurred for 317 days (33 % of the time). In fact, while the variant-unstratified $R^{G}$ only focuses on the total number of recorded infections, the variant-specific one detects whether any specific strain is growing. To better disentangle the behaviour of the variant-specific $R^{G}$, we investigated the period during which the Alpha variant has replaced the wild type (see Fig. 3B) by computing the national R for both strains. At the emergence of the Alpha variant in October 2020, the new infections caused by this strain followed a steady exponential growth. Nonetheless, these additional infections did not shift the total number of recorded infections until January 2021 (Fig. 1C). This is reflected in the national R of the Alpha variant, which immediately diverged from the variant-unstratified national R, which remained closer (and below the endemicity threshold) to the national R of the wild type, a waning strain, and only started growing above the endemicity threshold around March 2021.

One may observe larger increases for the first new variants (Alpha and Delta), at which emergence the $R^{G}$ skyrockets to values up to three times higher. Conversely, later variants are characterized by minor, but still relevant spikes. The variant-unstratified $R^{G}$ does not display this behaviour and settles around the endemicity threshold with only modest increases, a trend also shown by the local variant-unstratified R. The two time series (variant-specific and unstratified), however, converge as soon as a variant establishes as the only circulating strain, as seen during the first phase of the epidemic up to October 2020 and, briefly, around autumn 2021.

The epidemicity index (Fig. 3C) also holds a similar behaviour, with sudden spikes at the emergence of a new variant. Actually, the divergence between the variant-specific epidemicity index and the variant-unstratified one appears even wider than the discrepancy observed for R. This is apparent throughout the investigated timespan, but especially during the replacement of the wild type with the Alpha variant (Fig. 3D). Once again, the gap between the epidemicity index of the newer strain and that of the one being replaced proves wider than the difference between the national reproduction numbers of the two variants. A higher epidemicity index of the Alpha strain results from a lower epidemicity threshold (see Table 1). In addition, R takes higher values (Fig. 3B). Owing to a different generation time's distribution, the threshold reproduction number $R_{⋆}^{v}$ of the Alpha variant is much lower than that of the wild type, meaning that the control measure should target a much lower R to achieve unreactive conditions.

## 4 Discussion and conclusions

The emergence of new pathogenic variants during an epidemic induces shifts in the generation time distributions and in the values of reproduction numbers currently not accounted for by algorithms for the computation of epidemiological indices from data. Here, we have extended an existing sequential Monte Carlo algorithm for the real-time estimation of epidemiological indicators inclusive of variant-induced effects. Our main result is that adding such an additional layer of complexity is not a technicality nor a negligible and incremental effect, but rather a tool to provide significant corrections to the estimates of prognostic indices like e.g. the evolving values of the reproduction numbers from data. The epidemicity index is likewise susceptible to major corrections owing to the emergence of viral variants. The two metrics are traditionally used to assess the fate of an outbreak of infectious disease in the long vs. short term, and in the immediate aftermath of a variant's emergence. This has significant implications, in the authors' view, on effective epidemic control.

To that end, we computed the variant-specific local R (Trevisin et al., 2023). Our computations suggest that the R values of an emerging variant typically overshoot well above the endemicity threshold, while both R of the circulating strain and of the variant-unstratified one merely fluctuate around (or below) the critical threshold. This is the expected behaviour when the strain is replaced, as its disappearance is directly related to the condition R<1 being verified for a prolonged period. On the other hand, the behaviour of the variant-unstratified R only focuses on the total number of recorded infections. Thus, R proves insensitive to whether one strain has begun to grow until the emergent strain overcomes the previously circulating ones, and drives and controls the total number of infections.

This behaviour also emerged with the global index $R^{G}$, which accounts for all the variants spreading within a connected metapopulation. While the variant-unstratified $R^{G}$ showcases the same behaviour of the local R (being only dependent on the total number of infections), the variant-specific $R^{G}$ displays sudden spikes at the appearance of a new and evolutionary fitter strain. These jumps underline the presence of a variant growing more rapidly than the total number of infections, as was the case around November 2020 when the Alpha strain started replacing the wild type of SARS-CoV-2. This replacement went unnoticed in terms of the effective reproduction number until March 2021, when Alpha became the dominant strain and the variant-unstratified $R^{G}$ finally started growing above the epidemic threshold. The variant-specific $R^{G}$ anticipates this growth and highlights a potentially concerning situation well before a dominant strain is replaced. Failing to consider variants’ stratification may thus lead to serious underestimations of the severity of an epidemic.

The epidemicity index follows a qualitatively similar trend, and the variant-specific time series also diverges from the variant-unstratified at the emergence of newer and fitter strains. Similar spikes may be observed throughout the whole period studied. In addition to such spikes, the divergence between variant-specific and variant-unstratified time series appears substantially larger than the one observed with the reproduction numbers. This is a direct consequence of the higher reactivity of several strains than that of the wild type, characterized as it is by lower values of $R^{⋆}$ (Table 1). For this reason, epidemic controls should ideally not simply aim at achieving $R^{G}<1$, but rather $R^{G}<R^{⋆}$.

Our computational experiments are not devoid of limitations. First, to implement the framework introduced here, one has to gather data on, or analytically derive, the number of new infections caused by each relevant strain. While, in practice, the chance for a variant to be sampled might be slightly different from its actual prevalence among the sequenced samples, this probability is unlikely to swiftly change over time and impact the computation of R. Secondly, our framework requires knowledge of the variants' generation time distributions. This might prove challenging and in need of major assumptions at the emergence of a new variant (or at the emergence of the wild type of the pathogen) as reliable estimations of the serial interval and the generation time distribution usually require at least a few weeks of observations to be sorted out. As a result of its underlying algebra, the variant-stratified framework always provides more conservative estimates of the global epidemiological indices compared to the variant-unstratified ones. As such, even tentative estimates of the relevant generation time distributions can provide informative estimates of the values of the epidemiological metrics and help understand an evolving situation. Furthermore, strain replacement typically occurs in a few months, as suggested by the data shown in Fig. 1, and updated estimates of the generation time distributions may be used to adjust the inferred epidemiological numbers progressively. This implies that variant-specific frameworks point to the radically different description of the status of an outbreak – and its containment measures – than that of variant-unstratified ones. Inference of the variants' reproduction numbers may prove difficult during lull phases of the outbreak when the number of new infections for a specific variant is very low. While this is an established challenge for inference frameworks (Cori et al., 2013; Parag, 2021; Trevisin et al., 2023), working in discrete time allows one to exclude variants that do not achieve a steady prevalence or to aggregate data by adopting a larger time step, provided it is smaller than the mean of the generation time distribution. Working with a larger time step may thus increase the prevalence over a given time window and improve our sequential Monte Carlo's estimates of inferred reproduction number time series. However, we maintain that the framework put together improves the state of the art in this field.

In conclusion, our results show that whenever variants' emergence is considered, embedding the pathogens’ stratification into variants in algorithms for the computation of prognostic epidemiological indices produces substantially different results. In turn, this yields broad ramifications for optimal control measures. Our improved framework detects whether one specific strain grows more rapidly than the total number of infections and therefore foresees possible growths of new infections once the emerging variant becomes the dominant strain. Because our framework combines stratification in both space and variants, it provides a more precise estimate of the epidemiological metrics in a comprehensive environment. This is particularly relevant when outbreaks involve several communities and co-circulating pathogen strains. Finally, the sequential Monte Carlo process used here makes it possible to update the estimates of effective reproduction numbers and epidemicity indices as new surveillance data becomes available, with notable expediency. This framework may be applied to several practical scenarios, including when two or more airborne infectious diseases with similar infection routes (e.g., COVID-19, flu, etc.) circulate at the same time. It may be applied to any geographical setting, provided the data are available. Coupling this comprehensive framework with public health surveillance systems would create a pipeline that extends from data collection to the computation of the epidemiological indices, thereby securing updated indices that may be considered to plan control measures. Such integration would also make it possible to correct the values of these indices when needed, especially when data on variant prevalence is scarce, not collected daily, or needs frequent reassessments.

## CRediT authorship contribution statement

**Cristiano Trevisin:** Writing – review & editing, Writing – original draft, Visualization, Validation, Software, Methodology, Investigation, Formal analysis, Data curation, Conceptualization. **Lorenzo Mari:** Writing – review & editing, Visualization, Validation, Investigation, Formal analysis, Data curation. **Marino Gatto:** Writing – review & editing, Validation, Methodology, Investigation, Formal analysis. **Vittoria Colizza:** Writing – review & editing, Visualization, Validation, Investigation, Formal analysis. **Andrea Rinaldo:** Writing – review & editing, Validation, Investigation, Funding acquisition, Formal analysis, Conceptualization.

## Data availability statement

All codes and data involved in this study are available at the following Zenodo database https://doi.org/10.5281/zenodo.15011376 and are also available at the following public GitHub repository: https://github.com/cristianotrevisin/epidemiological-indices-metapopulation.

## Declaration

All authors declare no competing interests.

## Declaration of competing interest

The authors declare that they have no known competing financial interests or personal relationships that could have appeared to influence the work reported in this paper.
